# Evolutionary Traits and Genomic Surveillance of SARS-CoV-2 in South America

**DOI:** 10.1155/2022/8551576

**Published:** 2022-05-18

**Authors:** Pablo A. Ortiz-Pineda, Carlos H. Sierra-Torres

**Affiliations:** ^1^Laboratory of Molecular Biology and Genomics, InnovaGen Foundation, Popayán, Colombia; ^2^Human Genetics Laboratory, Department of Physiological Sciences, Faculty of Health Sciences, University of Cauca, Popayán, Colombia

## Abstract

Since the zoonotic event from which SARS-CoV-2 started infecting humans late in 2019, the virus has caused more than 5 million deaths and has infected over 500 million people around the world. The pandemic has had a severe impact on social and economic activities, with greater repercussions in low-income countries. South America, with almost 5% of the world's population, has reckoned with almost a fifth of the total people infected and more than 26% (>1/4) of the deceased. Fortunately, the full genome structure and sequence of SARS-CoV-2 have been rapidly obtained and studied thanks to all the scientific efforts and data sharing around the world. Such molecular analysis of SARS-CoV-2 dynamics showed that rates of mutation, similar to other members of the *Coronaviridae* family, along with natural selection forces, could result in the emergence of new variants; few of them might be of high consequence. However, this is a serious threat to controlling the pandemic and, of course, enduring the process of returning to normalization with the implicit monetary cost of such a contingency. The lack of updated knowledge in South America justifies the need to develop a structured genomic surveillance program of current and emerging SARS-CoV-2 variants. The modeling of the molecular events and microevolution of the virus will contribute to making better decisions on public health management of the pandemic and developing accurate treatments and more efficient vaccines.

## 1. Introduction

SARS-CoV-2 is a new single-stranded RNA virus that was reported to cause coronavirus disease (COVID-19) early in December 2019 and was declared a pandemic by the World Health Organization (WHO) in March 2020 [1]. This virus is actually causing the fourth wave of massive infections in several countries in which the most affected are the United States, India, Brazil, Russia, France, United Kingdom, Turkey, Argentina, Colombia, and Spain, according to WHO and worldwide news. By August 2021, WHO reported that more than 250 million people around the world had been infected with SARS-CoV-2 and more than 5.1 million people had died from COVID-19 (https://www.who.int/home). This pandemic is showing a significant adverse impact on social and economic activities and as expected, it has been most noticeable in several low-income countries (LICs) [2, 3].

Coronaviruses are enveloped positive-sense RNA viruses that contain the largest RNA genomes known to date within the *Coronaviridae* family [1, 4]. SARS-CoV-2 is a very close relative of the SARS-CoV virus, which caused its own epidemic between November 2002 and May 2004. The SARS-CoV epidemic resulted in 8,096 people infected and 774 people dead, with a fatality rate of between 10 and 16% [5, 6]. Another well-known family member is MERS-CoV, a virus responsible for the current epidemic of Middle East Respiratory Syndrome (MERS). Commonly known as camel flu, it began in 2012, infected 2,040 people, and showed a fatality rate estimated at 34% [7]. SARS-CoV, SARS-CoV-2, and MERS-CoV belong to the *Betacoronaviridae* subfamily. SARS-CoV-2 shares a 79.6% sequence identity with SARS-CoV [8] and 50% with MERS-CoV [9]. It is also notable that SARS-CoV-2 reveals a 96.2% homology with BatCov RaTG13, a bat coronavirus, which might suggest its zoonotic origins [8]. A hypothesis of an intermediate host mammal, the pangolin, was considered; but maximum-likelihood phylogenies and experimental approaches showed that the pangolin virus has a very low affinity for the human ACE2 receptor, suggesting a direct transmission from bats [10, 11].

Coronavirus ssRNA genomes are fast-changing entities with relatively high mutation rates in the virus superkingdom. Genetic surveillance and real-time tracking of such diversity are necessary because changes in the SARS-CoV-2 sequence are closely related to epidemiology impact: (1) the infectivity rate, (2) the mortality rate of infected people, and (3) the effectiveness of existing vaccines [12, 13].

In an effort never seen before in the history of science, the SARS-CoV-2 RNA genome was sequenced rapidly and in huge numbers to facilitate both diagnostic testing and genetic surveillance of the virus [14]. Additionally, studying the molecular aspects of the disease and the virus lets researchers understand the key molecular events that resulted in the pandemic, allowing them to make decisions and prognoses about what might happen to humans. This includes critical questions such as how is SARS-CoV-2 transmitted; how pathogenic is the virus in tertiary and quaternary spreading to humans; what are the mechanisms of virus shedding in asymptomatic and presymptomatic people; how COVID-19 should be diagnosed with effective and available procedures; how COVID-19 should be treated; what is the origin of SARS-CoV-2 and COVID-19 [12].

Genomic surveillance in Latin America is not only important but urgent, especially because the rate of infections is the highest proportional to the population. There are approximately 422 million people in South America, which roughly corresponds to 5% of the world population. Approximately 40 million out of the 250 million people infected are inhabitants of South America. This is more than 17% of infections worldwide and is the same number of infected people in India, a country with 1.37 billion people (https://www.worldometers.info/coronavirus/).

Another perspective to better understand the severity of the pandemic in South America is a comparison of the tested positive cases per million inhabitants. In Argentina (>116 k), Uruguay (>110 k), Brazil (>102 k), Colombia (>97 k), Chile (>90 k), Peru (>67 k), and Paraguay (>63 k), the prevelance of infection was 3 to 5 times greater than the proportion of infections in India (>23 k) and with approximately 30% of the corresponding population, according to the statistics from the World Health Organization (https://www.who.int/home). Of course, these high numbers of infectious rates are the result of the wrong, insufficient, and neglected policies administered in each country.

Latin America has limited economic resources not only in infrastructure but also in competent people to process, analyze, and decode these data from a public health perspective. In LICs, such limitations constrain the performance of genomic surveillance compared to those of developed countries [15–17]. These limitations are reflected in the 122,120 genomes reported to GISAID (global initiative on sharing all influenza data) from South America. This figure represents only 2.3% of the more than 5 million genomes reported; it should be noted that Brazil submitted 73,801 (61%) of the total.

In Colombia, over 8,000 genome sequences have been reported, representing 6% of all South American sequences and 0.14% of all GISAID sequences [18]. Venezuela, a country with limited epidemiological information, has only 172 sequences, corresponding to 0.14% of South American information and 0.0032% of world genomes. These gaps created blind spots for fully understanding the SARS-CoV-2 emerging diversity, limiting our ability to develop appropriate vaccines and efficient treatments.

The primary purpose of this review is to provide specific information about the sources of genomic diversity and the dynamics of viral microevolution that might help to understand the epidemiology and virulence of the SARS-CoV-2 and possible future coronaviruses. Moreover, we call for urgent genomic surveillance in Latin America, a region with particular SARS-CoV-2 dynamics currently spreading global and endemic variants that might have serious implications for virulence (pathology) and people's health.

## 2. Molecular Aspects and Microevolution of the SARS-CoV-2

According to the NCBI reference sequence (NC_045512.2), the SARS-CoV-2 RNA genome contains nearly 29,903 base pairs (bp) [19]. It is composed of 8 open reading frames (ORFs) and 4 structural genes: (1) the spike glycoprotein (*S*), typical of the *Coronaviridae* family, (2) the envelope protein (*E*), (3) the membrane protein (*M*), and (4) the nucleocapsid protein (*N*) distributed along the RNA genome and interlaced with the ORFs. Additionally, ORF1ab (21,555 nt) occupies 72% of the whole genome and encodes 16 nonstructural proteins (nsp) involved in proteolysis, replication, and adaptation to a new host [20]. The remaining ORFs (3a, 3b, 6, 7a, 7b, 8, 10) are accessory proteins involved in virion assembly but with functions still under investigation [21].

There are two main concerns about the source of variation in the SARS-CoV-2 genomes. First, we must understand that the more the population is infected, the higher the chances of finding a mutation are. As there are many copies of the virus, there is a proportional probability of a mutation occurring that would increase its virulence. This can be evidenced by the appearance of new variants in the middle of waves of infections in Europe, South Africa, the United States, Brazil, and now India [22–24]. The second aspect of concern is related to the natural process of emerging mutations. All DNA or RNA organisms are susceptible to nucleotide changes (substitutions, insertions, deletions, etc.) that could produce phenotypical changes [25]. Most of the changes are neutral (synonym mutations) and do not affect the phenotype. Some others might damage the viral structure or the capacity of the virus to reinfect, but natural selection gets rid of them. In contrast, the last few mutations are the ones that confer an adaptive response and make them dominant by the phenotype associated with them (i.e., more infectious) [26, 27]. For example, gaining affinity for the human angiotensin-converting enzyme 2 (ACE2) receptor opens the door for SARS-CoV-2 in human cells [28]. Humans are not immune to this source of variation; although the rate of change is much slower, some changes in the ACE2 gene translate into ACE2 protein polymorphism, which is now evidence of susceptibility to SARS-CoV-2 infection and COVID-19 severity [29–32].

In the year and a half since the virus has been spreading around the world, there have been reports and evidence that new variants are appearing around the world. According to the WHO and the US CDC (Centers for Disease Control), variations can be divided into three categories: (1) VOI (variants of interest), (2) VOC (variants of concern), and (3) VOHC (variants of high consequence) [33]. VOI are the ones with specific genetic markers that have been associated with changes in virulence. VOCs are variants for which there is experimental evidence of an increase in virulence (pathogenicity) and/or vaccine/treatment resistance. VOHC are the ones that pose a serious health threat, making available treatments useless [33]. Finally, the variants recently discovered with significant spreading capacities and with a potential menace are called variants under investigation (VUI). All of these variants are the result of evolutionary force imbalances that ponder how to increase diversity.

Genetic drift and selection pressures will limit the diversity of SARS-CoV-2 by limiting the virus's spread or survival due to random events or biosafety protocols. In contrast, the source of variation in any virus comes from mutation and recombination occurring in every event of infection and replication [25, 34].

Cumulative mutations in SARS-CoV-2 are directly correlated with five pandemic alarms: (1) an increase in disease severity and mortality risk; (2) the speed of transmission from human to human; (3) the effectiveness of available vaccines; (4) the effectiveness of diagnostic tests; (5) susceptibility to treatments [12, 35–37]. All these trepidations are examined under genomic surveillance, which consists of deep analysis to determine how sequence changes reflect variations in the phenotype that modify the mechanism to detect and neutralize the infection.

## 3. Nomenclature of SARS-CoV-2

Due to the fast spread of diversity observed in SARS-CoV-2, the need to name the lineages has emerged. This is evident in the nomenclature used by the main databases or organizations in charge of surveillance, like WHO, GISAID, and Nextstrain. Initially, variants were named according to the place where they were first reported (i.e., the UK, South Africa, Brazil, or India). A dynamic method was later proposed based on the use of a phylogenetic framework to track lineages that contribute the most to an active spread [38]. The two main databases for SARS-CoV-2 genomes and genomic epidemiology, GISAID and Nextstrain, respectively, settled on their own nomenclature [18, 39]. To make it easier and more practical to be discussed by nonscientific audiences, the World Health Organization recommended on May 30, 2021, using letters of the Greek Alphabet to denote variants of concern and interest [40].  Table 1 compiles lineages, the place where they were first identified, and important characteristics of variants. Until June 21, 2021, the group of VOCs was comprised of five variants: Alpha (UK B.1.1.7), Beta (South Africa B.1.351), Gamma (Brazil P.1), Delta (India B.1.617.2), and Omicron (South Africa B.1.1.529). Also, there are two VOIs under research: Lambda (Peru C.37) and Mu (Colombia B.1.621). Until now, no VOHC has been reported.

When comparing the variants, it is noticeable that a large number of all mutations are located in the spike (*S*) protein. Being the *S* protein of the coronavirus, the main determinant of host and tissue tropism, it is also the target of vaccines, neutralizing antibodies and inhibitors of virus entry [41, 42]. Furthermore, mRNA vaccines currently in use worldwide were designed to transcribe fragments of this protein. The *S* gene, which consists of 3,821 nucleotides coding for a protein of 1,273 amino acids, forms a trimeric spike (subunits S1, S2, and S2′) on the virion surface and plays an essential role in viral entry [43]. Coronaviruses may use two different pathways to enter the host cell: (1) the protease-mediated cell surface pathway and (2) the endosomal pathway [42, 44]. According to Hu and colleagues, the spike proteins of several coronaviruses are cleaved by host proteases in the S1 subunit for receptor binding and the S2 subunit for membrane fusion in the entry step of infection [29, 42]. Similar to SARS-CoV, the cellular receptor for SARS-CoV-2 is angiotensin-converting enzyme 2 (ACE2). However, the SARS-CoV-2 *S* protein has up to a 20-fold higher affinity for ACE2 than its counterpart, the SARS-CoV spike protein [28].

A recent study showed that a new serine protease (elastase-2) was introduced when a glycine replaced aspartic acid at position 614 of the spike protein [45]. Experimental studies have shown that it enhances viral replication in human lung epithelial cells and primary human airway tissues by increasing the infectivity and stability of virions. This is currently the dominant mutation in all VOC and VOI [26, 43, 46]. It is still uncertain whether the D614G (Asp614Gly) mutation affects the antigenic properties of protein *S*, although there is the possibility of positive natural selection. With the gain of transmissibility and the absence of preexisting immunity in the general population, the chances for this mutation to disappear seem remote, and it is not known whether SARS-CoV-2 is fully adapted for efficient growth in human cells [10]. Supported by robust sequencing and correlation with abundance, with reference to all sequences obtained at specific periods of time, one can determine if a new mutation is likely to become dominant.

## 4. The SARS-CoV-2 Mutation Rate

Because of the chemistry of the molecule, single-stranded RNA viruses have, on average, a higher mutation rate than DNA viruses. However, large coronavirus genomes such as SARS-CoV-2 are relatively stable thanks to a proofreading mechanism that operates during replication (nsp14 exonuclease) [10, 47]. Still, many mutations occur, generating stable variants like de D614G (Asp614Gly) that become dominant in about 6 months. Studies have shown that mutations occur more frequently in some regions of the SARS-CoV-2 genome [21, 48]. When measuring point mutations in the whole viral genome, Roy and colleagues determined the mutation frequency in *μ* = 9.4 × 10^−6^, which corresponds to 20,163 polymorphisms detected/(29,903 nucleotide genome size × 71,703 sequences analyzed in the dataset). When measuring only the nsp region (ORF1ab), the rate was 8.78 × 10^−6^. Within this region, the genes nsp1 and nsp2 have the highest rates of mutation at 1.12 × 10^−5^ and 1.08 × 10^−5^, respectively. These two are leader genes to inhibit immune response: nsp1 inhibits protein translation by blocking 40*S* ribosome and interferon (IFN) signaling, while nsp2 inhibits prohibitins 1 and 2 to disrupt the cellular environment [49]. Mutations in these two genes might confer novel viral outcomes to evade the host's immunogenic response.

Compared to other studies, the mutation rate in the SARS-CoV genome of family members was estimated to be 0.80–2.38 × 10^−3^ nucleotide substitutions per site/year, which is in the same order of magnitude as other RNA viruses when a time scale is involved [50]. For SARS-CoV-2, the moderate accumulation of changes observed in a year was approximately 6 × 10^−4^ nucleotides/genome/year [47]. Compared to those high rates, accessory genes ORF7a and ORF3a are 1.37 × 10^−5^ and 1.35 × 10^−5^ nucleotides/genome/year, respectively. ORF7a, an accessory protein, is thought to be involved in viral assembly or budding events specific to SARS-CoVs [49]. The accumulation of nonsynonymous mutations in this gene may provide new molecular options for increasing virulence efficiency [48]. On the other hand, ORF3a has been reported to have proapoptotic activity through mitochondrial damage and activating inflammatory responses of host cells [51]. The high rate of mutations in this gene could be interpreted as a devious strategy of the virus to finish its life cycle and kill the host cell. Genomic data can show how viral pathogens have responded to different forces of natural selection. In a model of codons, natural selection acting over any locus can be estimated using the proportion of nonsynonymous (dN) and synonymous (dS) mutations (dN/dS). Values of dN/dS > 1 can be interpreted as a positive natural selection because diversity arises. In contrast, negative selection or selective removal of the alleles that are deleterious results in dN/dS < 1 [48, 52].

When comparing genes within the SARS-CoV-2 genome, recent studies have found that all nsp genes (except nsp11), *S* (spike), and *M* (membrane) are under negative (purifying) selection (dN/dS < 1) [48]. This is reasonable because these are the genes (nsps, *S* and *M*) in which the host immune response is active. On the other hand, accessory proteins ORFs 3a, 6, 7a, 8, and 10, structural proteins *E* (envelope), and *N* (nucleocapsid) genes are under positive selection (dN/dS > 1), having different forms, arise in the possible new variants.

## 5. Mutations Related to VOC and VOI

To understand what is happening with the distribution of variants in South America, it is necessary to first understand what set of mutations is linked to each variant. Based on the SARS-CoV-2 genome data from GISAID, the platform Outbreak.info, updated daily, displays graphic information that correlates the presence of certain detected mutations with VOI and VOCs [46].

Similar to variant classification, mutations are also considered: mutations under observation (MUO), mutations of interest (MOI), and mutations of concern (MOC), sorted according to experimental observations in phenotypic changes related to virulence (pathogenicity) in SARS-CoV-2. We already mentioned D614G (Asp614Gly), a mutation now surviving in all VOI, VOC, and VUO. The current mutation of concern is *S*: E484K (Glu484Lys). A mutation of *G* > *A* in position 23,012 that changes a glutamic acid (*E*) for Lysine (*K*). This mutation is present in VOC Beta (B.1.351), Gamma (P.1), and Mu (B.1.621). This mutation increased dominance in Brazil [53] and recent studies have shown reduced neutralization by immune reactions, also called “escape mutations” [54, 55], and higher infectivity when in the presence of other mutations [56].

**Figure 1:**
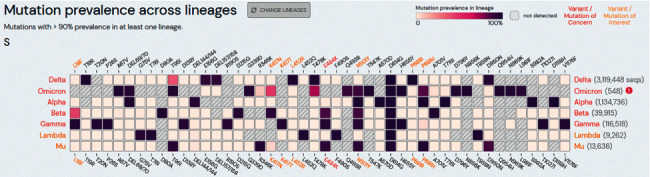
Cross reference of variants of concern (VOC) and (VOI) variants of interests (*Y*-axis) and the occurrence and frequency of point mutations (*X* axis). Mutations and frequency were measured with >90% prevalence of that specific mutation in at least one lineage. Text in red represent variants/mutations of concern. Yellow: variants/mutations of interest. White text: variants/mutations under surveillance or observation. Graphic modified from outbreak.info website [46] and customized to show only the VOC and VOI currently spreading in South America. By the time of manuscript writing, a few cases of Omicron were present in South America but reached over 10% in South Africa and over 3% in the fourth wave of infections in Europe in the last month. Source: outbreak.info/GISAID.

The following mutations are all of interest (MOI), but their impact resides in the synergic functionality with other mutations. K417N (Lys417Asn) and K417T (Lys417Thr) mutations are *G* > *T* substitutions at position 22,811 and *A* > *C* substitutions at position 22,810, respectively, that change a Lysine (*K*) for Asparagine (*N*) or Threonine (*T*) and have been found in Beta, Gamma, and Omicron variants, and appear to have higher rates of infectivity [56]. Another mutation under the radar is N501Y (Asn501Tyr). This causes an *A* to become a *T* at position 23,063, resulting in asparagine (*N*) for tyrosine (*Y*). This mutation is present in Alpha, Beta, Gamma, Omicron, and Mu. This last variant was first identified in Colombia and rapidly spread in the northwest region of South America. It is involved in higher infectivity [56] and, mechanistically, the N501Y (Asn501Tyr) substitution improved the affinity of the viral spike protein for cellular receptors, and researchers have suggested this mutation be classified as a MOC [57]. To easily visualize MOCs and MOIs with each VOC and VOI, Figure 1 shows the variants currently active in South America and their respective mutations and frequency. Additionally, Table 2 shows the frequency of each variant in every continental country in South America.

## 6. Variant Distribution in South America

To have a broader perspective of what is happening in South America, we can examine the distribution and frequencies of the variants currently identified. Table 2 shows the SARS-CoV-2 variants (VOC and VOI) and frequencies in South American countries together with the amount (number and percentage) of sequences submitted to GISAID by the end of September of 2021. GISAID data shows Delta as the largest widespread, with an almost 90% frequency of all recently sequenced variants [18]. Delta increased rapidly from 1% in early June to 19% in late July to 90% in early November. This variant is known for the mutations in the Spike gene: T19R (Thr16Arg), T95I (Thr95Ile), L452R (Leu452Arg), T478K (Thr478Lys), D614G (Asp614Gly), P681R (Pro681Arg), and D950N (Asp950Asn). Similar to variant Mu, they share the mutations T95I (Thr95Ile) and D950N (Asp950Asn) that seem to correspond to the significant increase in infectivity. No data on the severity of the disease, mortality, or vaccine resistance have been linked to these latter mutations yet.

In second place is the Gamma variant, with an almost insignificant 5% compared to the 85% it had in early July. It was first identified on January 6, 2021, in Tokyo, Japan, by travelers from the Brazilian Amazonia [58]. It is currently in every continental country in South America. It contains 14 mutations, 10 of which are in the *S* gene and 5 of those 10 are the mutations: L18F (Leu18Phe), K417T (Lys417Thr), E484K (Glu484Lys), N501Y (Asn501Tyr), and D614G (Asp614Gly). It had reached 96 countries by the end of June 2021. Recent studies have shown a significant reduction in the neutralization of Pfizer and Moderna vaccines in fully dosed people [59], and the efficacy of Sinovac vaccines is close to 50%, but exact measurements are still being conducted in studies [60].

The third most frequent variant is shared by Lambda (C.37, Nextstrain: 21G) and Mu (B.1.621, Nextstrain: 21H), which are about 3 to 5% frequent each. Lambda (C.37), also known in Latin America as “Variante Andina,” was originally reported in Peru in August 2020 and classified by WHO as VOI on June 14, 2021. The Lambda variant contains six nonsynonymous mutations: G75V (Gly75Val), T76I (Thr76Ile), L452Q (Leu452Gln), F490S (Phe490Ser), D614G (asp614Gly), and T859N (Thr859Asn) in the *S* gene, plus a novel deletion (Δ246–252) [61]. Interestingly, by early May of 2021, the Lambda variant (C.37) was close to 93% of the frequency in Peru and reached over 30 countries, including Australia [62]. It is still profuse in 1 out of every 5 cases in Argentina, Bolivia, and Chile. It is not clear if the Lambda variant has implications for vaccine resistance or implications for disease severity, as was speculated in the news.

The other 3% is Mu. It is an outlier of distribution because the variant was found mostly in Colombia, where it counts for 95% of the newly reported cases in June and July 2021 [63]; 3% in other South American countries; 2% in the Caribbean islands and the rest of the world where it spread. Inside Colombia, it is still near 9% but has been outspread by the Delta variant. In May 2021, the Mu variant represented 95% of Colombian sequences, and by early August, there was still over 82% [17, 63]. No evidence of the severity of the disease or symptoms has been reported yet.

The thriving capacity of SARS-CoV-2 to mutate has resulted in, in some cases, convergent evolution and a boost of mutations that are not phylogenetically related. This is, the same mutations are emerging in different places [47]. As with D950N (Asp950Asn), the mutation *G* > *A* at position 24,410 converts an aspartic acid (*D*) to an asparagine (*N*). It surfaced almost simultaneously in opposite places of the world: the Delta variant (B.1.617.2) in India and the Mu variant (B.1.621) in Colombia. Studies have shown that in the *S* gene alone, there is one location with at least 15 recurrent mutations, suggesting convergent evolution and a particular interest in the context of adaptation of the virus to the human host [47].

Although these data compilations reveal high mutation rates and pressures of selection in favor of the arising diversity of SARS-CoV-2, it is important to mention that these data must be interpreted carefully because some statistical metrics might technically be biased in different ways. On the other hand, the majority of sequences in South America come from just a few research centers or government-funded laboratories in which the collected samples are typically from main urban areas and might not represent the full diversity, especially from regions with difficult access or those near to frontiers with other countries. Secondly, the sequences are being obtained from different platforms. It is well known that PacBio, ION Torrent, and Oxford NanoPore technologies offer longer reads at a quality cost, making them difficult to compare with Illumina or Sanger methods. This highly error-prone platform might show an inaccurate reading of nucleotides as possible point mutations if replicates and controls are not rigorous enough. Third, countries in South America have the lowest speed for updating genomic information in GISAID. By the time this article was written, there was no information about the last three months from Bolivia, Uruguay, Paraguay, and Venezuela.

As a fourth aspect, most of the statistics to measure transmissibility are based on confirmed positive cases; however, only in Germany, studies showed that almost 42% of people are unaware of their current status of infection [64]. An unmeasurable number of people in South America are infected but not officially diagnosed, therefore making the fatality of SARS-CoV-2 much lower and the transmissibility much higher [65].

And finally, these molecular dynamics of mutation and variant frequencies are time-sensitive and a time interval as low as 30 days is enough to significantly change the epidemiological landscape. According to GISAID, South American countries show a median of 92 days (equal to 3 months with a range of 27 to 271 days) for the deposition of sequences [18]. Compared to the 16 days of developed countries in Europe, South American countries are taking too much time to reach the correct epidemiological and appropriate public measures based on evidence to control the pandemic, thus resulting in massive waves of infection and new variants appearing.

## 7. Conclusions

This compiled data present irrefutable evidence that Latin America is a special case of the genomic dynamics of SARS-CoV-2. The distribution of all mutations found accounts for over 63% of the whole SARS-CoV-2 genome, representing a high degree of polymorphisms, with mutation and positive natural selection as sources of diversity. This was possible to understand as scientific collaborations were based on data sharing through public databases like GISAID.

However, Latin American reports are somehow incomplete and biased because there are not enough genome sequences generated in proportion to the rates of infection; and the small amount of data reported does not represent all the territory. Additionally, the limited access to samples in geographically challenging locations restrains real-time monitoring of variants' distribution.

Continuous genomic surveillance to track existing VOC and VOI and the rapid emergence of new variants is critical for guiding and making appropriate public health decisions aimed at limiting the spread and finding effective treatments. Additionally, the phylogenetic information in the evolutionary perspective will give us keys to creating models to predict new epidemics of coronavirus, as has been happening approximately every decade since SARS-CoV in the early 2000s.

This pandemic teaches us that a successful exit from these worldwide circumstances lies in global policies and contributions from all countries. Finally, Latin America urges the world to approach higher standards in technology and human talent to overcome the limitations that are currently happening to track, in real time, the dynamics of COVID-19 and SARS-CoV-2. An immediate solution could be that countries with robust surveillance programs and NGS infrastructure, like Brazil, Chile, and Colombia, start to work with samples provided by neighboring countries to homogenize and keep up the upgraded information with the help of the Pan American Health Organization.

## Figures and Tables

**Table 1:** Nomenclature of SARS-CoV-2 variants and noticeable pathological characteristics.

WHO label	Pango lineages	GISAID	Nextstrain	First discovered	Increased		
					Infection	Severity/lethality	Vaccine resist
*Currently designated variants of concern*							
Alpha	B.1.1.7	GRY	20I (V1)	UK, Sep-2020	Yes >20%	Yes >50%	
Beta	B.1.351	GH/501Y.V2	20H (V2)	South Africa, May-2020	Yes >50%		Reduced
	B.1.351.2−						
Gamma	P.1	GR/501Y.V3	20J (V3)	Brazil, Nov-2020	Yes >50%	Possibly
	P.1.1–2					Increased
Delta	B.1.617.2	G/478K.V1	21A	India, Oct-2020	Yes >60%	Yes >50%	Reduced
	AY.1/2						
Omicron	B.1.1.529	GR/484A	21K	Multiple countries, Nov-2021	UI∼>60%	UI	UI

*Currently designated variants of interest*							
Lambda	C.37	GR/452Q.V1	21G	Peru, Dec-2020	UI	UI	UI
Mu	B.1.621	GH	21H	Colombia, Jan-2021	UI	UI	UI

*Currently designated alerts for further monitoring*							
Epsilon	B.1.427	GH/452R.V1	21C	United States, Mar-2020	Yes >15%		Possibly
	B.1.429^*∗*^						Reduced
Zeta	P.2^*∗*^	GR/484K.V2	20B	Brazil, Apr-2020	UI		
Theta	P.3∗	GR/1092K.V1	21E	Philippines, Jan-2021	UI		
	R.1	GR	—	Multiple countries, Jan-2021	UI		
	R.2						
	B.1.466.2	GH	—	Indonesia, Nov-2020	UI		
	AV.1	GR	—	UK, Mar-2021	UI		
	B.1.1.318	GR	20B	Multiple countries, Jan-2021	UI		
	B.1.1.519	GR	20B	Multiple countries, Nov-2020	UI		
	AT.1	GR	—	Russia, Jan-2021	UI		
	B.1.214.2	G	—	Multiple countries, Nov-2020	UI		

UI: under investigation. Our own source. Data from OMS, CDC, ECDC, and Outbreak.info.

**Table 2:** Distribution and frequencies of VOC and VOI in continental South America.

		VOC				VOI		N %	
NextStrain⟶		20I	20H	20J	21A	21G	21H		
GISAID⟶		GRY	GH	GR	G	GR	GH		
Pango⟶		B.1.1.7	B.1.351	P.1	B.1.617	C.37	B.1.621		
Place⟶		UK	SA	Br	In	Pe	Col		
Country\WHO⟶	wt+	*α*	*β*	*γ*	*δ*	*λ*	*μ*		
Argentina				26%	42%	32%		11.156	8.2%
Bolivia^*∗*^	24%			52%		24%		155	0.1%
Brazil				1%	99%			83.947	61.4%
Chile					94%		6%	16.345	12.0%
Colombia					92%		8%	8.667	6.3%
Ecuador					99%		1%	3.552	2.6%
Paraguay^*∗*^					100%			887	0.6%
Peru					100%			11.072	8.1%
Uruguay^*∗*^	100%							739	0.5%
Venezuela^*∗*^				100%				189	0.1%
South America				5%	89%	4%	2%	136.709	100%

Clades as headers. *α* = alpha, *β* = beta, *γ* = gamma, *δ* = delta, *λ* = Lambda, *μ* = Mu. wt+ = “wild-types”/original variants. N: number of sequences curated in GISAID (29 Nov 2021). Analysis on November 2021, UK: United Kingdom. SA: South Africa, Br: Brazil, In: India, US: United States, Pe: Peru, and Col: Colombia. At the time of manuscript writing, isolated cases of Omicron were reported in Brazil. Countries with ^*∗*^ data not available from the last three months. Our own source. Data from: Nextstrain.org.
